# Relationship between body image, anxiety, food-specific inhibitory control, and emotional eating in young women with abdominal obesity: a comparative cross-sectional study

**DOI:** 10.1186/s13690-021-00526-2

**Published:** 2021-01-25

**Authors:** Zhong-Hua He, Ming-De Li, Chan-Jun Liu, Xiao-Yue Ma

**Affiliations:** School of Journalism and New Media, Xi’an Jiaotong University, Xi’an, China

**Keywords:** Body image, Anxiety, Food-specific inhibitory control, Emotional eating, Abdominal obesity, Young women

## Abstract

**Background:**

Abdominal fat deposition is a key component of obesity, which is associated with an increased risk for a number of mental disorders. The current study aims to explore the relationship between body image, anxiety, food-specific inhibitory control, and emotional eating in young women with abdominal obesity.

**Method:**

A total of 224 participants were recruited: 168 were non-abdominal obesity and 56 were abdominal obesity. Participants completed the following questionnaires and behavioral tests: the Body Mass Index (BMI) -based Silhouette-Matching Test (SMT), the State-Trait Anxiety Inventory (STAI), Food Stop Signal Task (SST), the Emotional Eating Scale (EES).

**Results:**

Abdominal obesity women had significantly higher levels of trait anxiety, cognitive difference, expectational difference in body image but lower self-reported emotional eating level compared to the control group. Anxiety mediated the relationship between cognitive difference of body image and depression _eating_ in young females with abdominal obesity. In addition, only among abdominal obesity individuals, expectational difference of body image were significantly and positively correlated with food-specific inhibitory control and trait/state anxiety.

**Conclusion:**

The findings suggest it is of critical importance to promote a healthy body image recognition and expectation and improve mood regulation for young females with abdominal obesity high in trait anxiety.

## Background

Abdominal fat deposition is a key component of obesity [1]. Some studies have shown that abdomen circumference (AC) may be a better predictor for the risk of type 2 diabetes, medical care costs, and all-cause mortality than body mass index (BMI) [2–5]. Abdominal obesity are characterized with excessive body fat in abdomen circumference [6]. The prevalence of visceral fat that accumulates around abdominal organs is increasing worldwide [7, 8]. Visceral fat cells have an crucial impact on overall health and well-being. Inappropriate diets such as high-calorie foods are other main influential risk factors for increasing abdominal obesity. Consequently, AC has emerged as a candidate for assessing abdominal obesity. In fact, AC has been reported to predict mortality risk better than BMI [9]. The AC is positively correlated with the abdominal fat. Hence, the AC is a valuable, convenient and a simple measurement method which can be used for identifying the individuals who are at an increased risk for the above mentioned diseases. The identification of the abdominal obesity by the abdomen circumference measurement is easily accessible and should become the obligatory part of any physical examination [10]. AC, frequently used as a simple, inexpensive measure of central obesity in population-based studies, has been shown to be associated with depression in some studies [11].

Eating behaviors, negative emotions (i.e., anxiety and angry), inhibitory control, and body image perception are implicated in the multifactorial psychological factors of obesity [12–16]. Obesity has been associated with an increased lifetime risk for major depression and panic disorder or agoraphobia, particularly among females [17]. They always use food to cope with stress and emotions. Eating has been recognized as a coping mechanism for alleviating and dealing with stress and emotions [18] by either undereating or overeating [19].

Body image has been demonstrated to be associated with obesity either as a cause or as a result that impacts on weight control behaviors [20, 21]. Perceptual body size misperception (either underestimation or overestimation) occurs more in individuals with greater obesity, and it can potentially lead to a lesser awareness of the health risks associated with obesity and a reduction in the implementation of weight control behaviors such as dieting [22].

Altered inhibitory control has been implicated in obesity. Several studies concluded that obesity and binge-related eating disorders (EDs) are associated with poor inhibitory control [23, 24]. The stop signal task (SST) was chosen as it is commonly used to assess motor control, with cognitive underpinnings that are clearly established and may have relevance to eating and weight-control behaviors (e.g., cognitive control exercised when resisting urges to eat) [25]. However, in both adults and youth, the findings were largely inconsistent. Three studies reported greater SSRTs (Stop Signal Reaction Time) in obese adults and overweight individuals [26, 27]. In contrast, some studies found no overall differences in SSRT between normal weight groups and overweight/ obese adults [26, 28–31].

Emotional eating has been observed in both obese individuals [32] and a critical review of the literature concluded that there is no relationship between body mass index and emotional eating [33]. Thus, vulnerability to emotional eating does not appear to be simply a function of weight. Emotional eating is likely effected by other psychological factors. Dysfunctional eating behaviors appeared to correlate strongly with body dissatisfaction, and perfectionism in girls [34]. In addition to body image, individual differences in affective traits and states may account for some of the observed variability in the effects of emotions on eating [35].

Several studies have found that obesity were more concerned about their physical appearance (body dissatisfaction and obsession with being thin) [36]. In fact, some authors argue that these types of cognitive variables could explain the vulnerability shown in people who go on to develop an eating disorder [37]. Likewise, common mental disorders were associated with an increased risk of obesity, and that the risk of obesity increased with the number of episodes of anxiety [38]. To be more specific, several studies have shown that youths who feel bad physically also feel bad emotionally [39]. Jansen et al. [40] reported that obese individuals high in negative affect consumed more food-specific than individuals low in negative affect following a negative mood induction, relative to a neutral mood induction. In contrast, lean individuals consumed comparable amounts of calories in the negative and neutral mood induction conditions, regardless of their level of negative affect [40]. With regard to this question, Braet et al. [41] point out that different psychological mechanisms and patterns seem to be in place in obesity female versus the control group. Considering the conclusion that high trait anxiety was positively associated with food-specific intake for obese individuals, but not their lean counterparts [35], it was possible to infer that the obesity group is more vulnerable to developing eating disorders, as those obesity female generally show more body image dissatisfaction, worse food-specific inhibition, and higher levels of anxiety regarding their body and weight and follow unhealthy diet administration. Therefore, anxiety is inferred to be the psychological mechanisms and patterns of of inappropriate emotional eating in obese youths [42].

More than half of the females preferred their ideal figure to be underweight, whereas about 30% males chose an overweight figure as their ideal model. Females were generally more concerned about body weight, body shape and eating than males. As suggested in the aforementioned studies, obesity female may be significant risk conditions, especially in youth, associated with inappropriate weight-control behaviors, emotional distress (anxiety, depressive symptoms, etc.) and concerns about one’s own body image (body dissatisfaction, negative beliefs about one’s body and eating, etc.) [43]. In addition, in China, despite having high obesity prevalence rates and more serious abdominal obesity prevalence rates [44–46], no studies have been carried out to analyse body image dissatisfaction, inappropriate emotional eating behaviors, food-specific inhibitory control and their relationship with variables of emotional distress according to whether they are abdominal obesity. In addition to state anxiety, trait anxiety may also be a risk factor for emotional eating among obese individuals [35].

Although the prevalence of overall obesity as measured by BMI is well-documented and it has increased dramatically in the past 2 decades [47, 48], little is known about the psychological characteristics of abdominal obesity in young women. Taking these into account, The present study established the following objectives: (a) to compare the level of inhibitory control, emotional eating, anxiety and body image between abdominal obesity and non-abdominal obesity in young women, (b) to compare abdomen circumference -related differences in relation between the level of inhibitory control, emotional eating, anxiety and body image in abdominal obesity and non-abdominal obesity groups, (c) to test the hypothesis that anxiety is the psychological mechanisms and patterns of of inappropriate emotional eating in obese youths.

## Methods and materials

### Study design

A comparative crossectional study approach was used.

### Participants

Xi’an was the place where this study was conducted. It is an important central city in western of China. The study was run beginning from May 2th, to October 20nd, 2019. All the participants were recruited through advertisements at local universities. They were selected by convenient sampling to participate in anonymous questionnaires survey and behavioral experimental tasks. The inclusion criteria were; being aged 18–25. In this study, the term ‘young women’ refers to the study population at hand with individuals aged 18–25. Young women were excluded from the study when the target individuals had a serious handicap, visual impairment, chronic neurological disorder (e.g., mental retardation), or psychiatric disorder.

The sample consisted of 224 young women who signed an informed consent. Of the 224 participants, 25% were abdominal obesity individuals (Non-abdominal Obesity: 168, abdomen circumference ≤ 85 cm; Abdominal Obesity: 56, abdomen circumference ≥ 85 cm) [49]. They studied in college in Xi’an city and received small presents for their participation. None reported significant medical impairments or physical and mental illness.

### Procedure

These female youth accepted tests in a quiet room individually. They completed tasks in a fixed order: demographic, height, weight and body image perceptions (the body mass index -based Silhouette-Matching Test), anxiety (state-trait anxiety inventory), Food- inhibitory control (the Food- Stop Signal Task), emotional eating (the Emotional Eating Scale). Each measure is described in detail in the following section.

### Measures

#### Body image perceptions

**Fig. 1:**
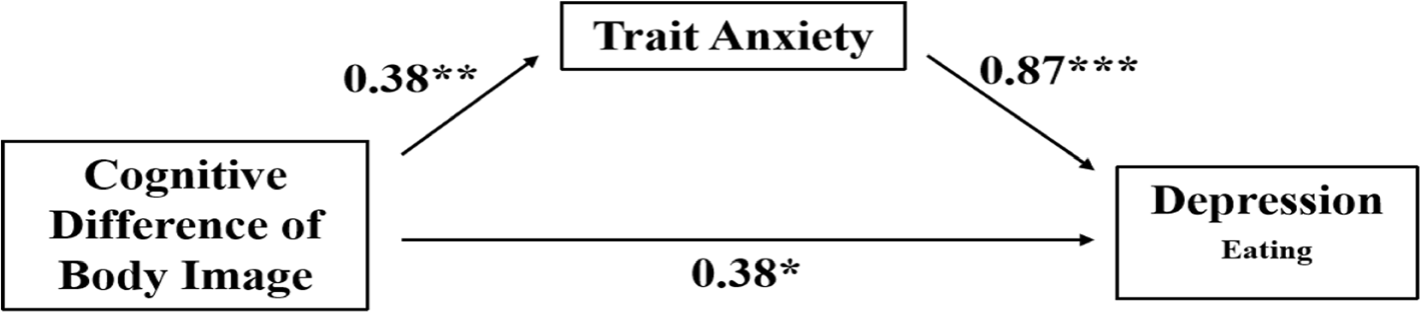
Trait anxiety mediation effects in the relationships between cognitive difference of body image and depression _eating_ in the female abdominal obesity group controlling for age, BMI, and family SES. Significant paths from cognitive difference of body image to depression _eating_ (using bias-corrected bootstrapped confidence intervals). Mean path coefficients were obtained using 2000 bootstrap samples. *Note.* *p < .05. **p < .01.***p < .001

Body image attitude was measured by the body mass index (BMI)-based Silhouette-Matching Test (SMT; [50]). In this test, participants were presented with silhouettes of figures ranging from very slim to very full, and were required to choose a number below the figures (1 to 27) to indicate (a) their current figure, (b) the ideal figure which they would like to have. In the current study, participants’ selection of the current figure was significantly correlated with current BMI, as calculated from a participant’s reported height and weight (r = .74, *p* < .001, df = 224). The degree of body image dissatisfaction was quantified with two measures: (1) Cognitive difference of body image (Cognitive 27-point SMT-BMI): the absolute value of this difference as some individuals may prefer a fuller image of themselves; and (2) Expectational difference of body image (Cognitive 27-point SMT-Expectational 27-point SMT): the difference between current and ideal body image (subjective drive for thinness). Similar methods have been used previously [51].

#### Anxiety

Levels of anxiety were measured with the Chinese version of the State-Trait Anxiety Inventory (STAI) S-Anxiety Scale [52, 53]. There are 2 subscales within this measure. The State Anxiety Scale (S-Anxiety) evaluates the current state of anxiety, asking how respondents feel “right now,” using items that measure subjective feelings of apprehension, tension, nervousness, worry, and activation/ arousal of the autonomic nervous system. The Trait Anxiety Scale (T-Anxiety) evaluates relatively stable aspects of “anxiety proneness,” including general states of calmness, confidence, and security. The STAI has 40 items, 20 items allocated to each of the S-Anxiety and T-Anxiety subscales. All items are self-rated on a 4-point scale of 1 = not at all, 2 = somewhat, 3 = moderately and 4 = very much, and they are added to obtain a total score for each respondent. Total scores range from 20 to 80, with higher scores indicating higher levels of anxiety. The scale has been tested and validated for the Chinese context. The Cronbach’s alpha level was acceptable (.82 and 0.79).

#### Food- inhibitory control

In the Food-stop signal task [27, 54], participants need to classify continuously presented stimuli according to simple criteria by key press. A stop signal is presented unpredictably in a random subset of trials following the display of the imperative stimulus and before the anticipated response. Participants are instructed not to execute the response in these trials. The paradigm is based on the theoretical horse-race model 55], which assumes independent go- and stop-processes. If the stop-process terminates before the go-process, a response is effectively inhibited. Good stopping performance is reflected in a fast stop-process which can be initiated late and which will, therefore, still terminate an ongoing response tendency. Participants were instructed to select as quickly and accurately as possible on which side the picture appeared by pressing either a left or a right response button. In the low calorie and high calorie food- stop signal task, there were respectively 3 kinds of photographs of shapes (e.g., quadrate, roundness, etc.), low-calorie food items (e.g., tomato, apples, etc.) and high calorie food items (e.g., fried chickens, desserts, etc.). A picture appeared in either on the left or right side of the fixation cross. A left or a right response button respectively represents a response to specific stimulus. In stop trials, a small blueberry /doughnut appeared over the picture as the stop signal after a variable delay. Upon any response detected or after 1500 ms, the screen was cleared. The recorded scores reflected “stop” accuracy in stop trials. The dependent variable, stop signal reaction time (SSRT), was calculated by subtracting the mean stop delay from mean reaction times. Higher SSRTs indicate decreased inhibitory control. Higher stop signal accuracy (SSACC) indicated better performance.

#### Emotional eating

The emotional eating scale [56] has been used to investigate eating behavior in response to negative emotions. We used the Chinese version of the Emotional Eating Scale [57], which contains four factors: eating in response to anxiety (four items), depression (nine items), anger/hostility (five items) and positive emotion (five items). Participants were asked to respond to questions about their desire to eat when experiencing certain emotions (e.g., “Do you have a desire to eat when you are sad, irritated, worried, or lonely?”) on a 5-point Likert scale ranging from 1 (never) to 5 (very strong). Higher scores indicate a stronger desire to eat. In the current study, Cronbach’s α was 0.73(anxiety) -0.80 (anger/hostility) for each emotional eating scale.

### Covariates

We adjusted for age, BMI, family socioeconomic status (SES). Although ideally, all participants should have provided information including all three components (education, occupation, and income) of SES, the majority of students, especially in China, only know and are more willing to provide information about education and occupation than income data [58]. Therefore, our study only considered the education and occupational prestige of participants’ parents. These were assigned based on categories of the Hollingshead Index matched as closely as possible to modern education and occupation status [59]. The family SES composite index was the sum value of parents’ SES.

### Statistical analysis

The descriptive statistics, correlations and path analysis were computed using SPSS 16.0 and AMOS 7.0 software [60]. Depending on the scale of the variable, the mean, standard deviations, and proportions were presented as a descriptive summary. And a comparison of the abdominal obesity and non-abdominal obesity groups was conducted through independent-samples *t* test. Two separate correlation analysis were run. Partial correlations was used to assess whether there are association differences of inhibitory control, emotional eating, anxiety and body image between abdominal obesity and non-abdominal obesity in young women.

Mediation tests indicated whether the association between two variables resulted from another variable or a set of variables. Path analysis is a specific tool of the structural equation model (SEM) analysis to analyze assumed relationships of multivariate data. Mediation tests were computed using path analysis in AMOS 7.0 with maximum likelihood estimation to examine the significance of the direct effects of cognitive difference of body image on depression _eating_ in the female abdominal obesity group, mediated through the anxiety. The structural equation model as illustrated (see Fig. 1) was tested for indirect effects of anxiety with a bias-corrected bootstrapping procedure based on 2000 bootstrap samples to estimate standardized regression estimates. For a good fit, the degrees of freedom (χ^2^/df) should be as small as possible, and values less than three indicate a good or acceptable fit. Goodness of fit indices were assessed based on following criteria: Tucker Lewis index (TLI) and comparative fit index (CFI) close to 0.9 and Root Mean Square Error of Approximation (RMSEA) < 0.8 [61].

## Results

### Body image, anxiety, food- inhibitory control and emotional eating

Cognitive difference and expectational difference of body image were greater than zero. All the participants (100%) perceived themselves to be fatter than physical truth and wanted a more slender figure. Participants of both groups were equivalent in age. However, no significant difference between the groups with respect to the stop signal accuracy (shape, low calorie, high calorie food), the stop signal reaction time (shape, low calorie, high calorie food), depression _eating_ and anger/hostility _eating_. Abdominal obesity female youths reported higher levels of cognitive difference and expectational difference of body image (*t* = − 2.05, *p* < .01; *t* = − 11.19, *p* < .001), while non-abdominal obesity ones reported higher emotional eating scores about anxiety _eating_ and positive emotion _eating_ (*t* = 3.46, *p* < .01; *t* = 4.34, *p* < .01) (Table 1).

**Table 1:** Body Image, Anxiety, Food- inhibitory Control and Emotional Eating of Two Abdomen Circumference Groups

	Non-abdominal Obesity (*n* = 168)	Abdominal Obesity (*n* = 56)	*t* value	*p* value
Age	20.56 (0.86)	21.02 (1.02)	0.76	0.40
BMI	20.44 (1.95)	25.33 (0.93)	−14.26	***
Family SES	54.19 (18.27)	60.71 (27.87)	−0.20	*
Body Image _CD_	6.13 (2.88)	7.03 (2.63)	−2.05	**
Body Image _ED_	4.07 (3.74)	8.14 (1.65)	−11.19	***
Trait Anxiety	38.00 (7.90)	40.36 (8.08)	−1.82	0.07^†^
State Anxiety	40.62 (8.43)	42.79 (8.26)	−1.77	0.08^†^
SSACC _Shape_	0.52 (0.06)	0.53 (0.05)	−0.97	0.34
SSRT _Shape (ms)_	505.23 (80.62)	489.31 (85.19)	1.26	0.21
SSACC _Low Calorie_	0.58 (0.06)	0.59 (0.05)	−1.00	0.32
SSRT _Low Calorie (ms)_	581.27 (85.91)	597.50 (89.51)	−1.21	0.23
SSACC _High Calorie_	0.57 (0.06)	0.57 (0.06)	−0.57	0.57
SSRT _High Calorie (ms)_	569.63 (84.23)	580.77 (96.51)	−0.77	0.41
Depression _Eating_	2.15 (0.69)	2.19 (0.89)	−0.28	0.78
Anxiety _Eating_	1.76 (0.48)	1.50 (0.53)	3.46	**
Anger/Hostility _Eating_	1.73 (0.54)	1.60 (0.52)	1.62	0.11
Positive emotion _Eating_	3.44 (0.86)	3.01 (0.55)	4.34	**

*Note*. *SES* Socioeconomic status, *CD* Cognitive Difference, *SSACC* Expectational Difference; the stop signal accuracy, SSRT the stop signal reaction time; ^†^*p* < .10. *p<.05.**p< .01.***p < .001

### Partial correlations between body image, anxiety and the stop signal task

Partial correlation analysis showed that for abdominal obesity female youths (abdomen circumference ≥ 85 cm), the correlation between expectational difference of body image, trait anxiety, state anxiety, SSACC _Low Calorie_, and SSACC _High Calorie_ were significant (*r* = .59, *p* < .001; *r* = .47, *p* < .001; *r* = .32, *p* < .05; *r* = .40, *p* < .01;). For non-abdominal obesity ones (abdomen circumference ≤ 85 cm), the correlation between cognitive difference of body image, SSACC _Low Calorie_, and SSACC _High Calorie_ were significant (*r* = .18, *p* < .05; *r* = .23, *p* < .01). However, the correlation between expectational difference of body image, trait anxiety, SSACC _Low Calorie_, and SSACC _High Calorie_ were not significant. For non-abdominal obesity ones, the correlation between body image and most emotional eating scores were negatively correlated. On the contrary, in the abdominal obesity group, the correlation between cognitive difference of body image, expectational difference of body image and depression _eating_ were positively correlated (*r* = .41, *p* < .01; *r* = .28, *p* < .05) (Table 2).

**Table 2:** Partial correlations between body image, anxiety, the stop signal task (SST) and emotional eating controlling for age, BMI, and family SES

Variables			Non-abdominal Obesity		Abdominal Obesity	
	*Body Image*		*Body Image*		*Body Image*	
	Cognitive Difference	Expectational Difference	Cognitive Difference	Expectational Difference	Cognitive Difference	Expectational Difference
Trait Anxiety	.010	.16*	−.03	.08	.43**	.59***
State Anxiety	.15*	.25***	−.07	−.01	.37**	.47***
SSACC _Shape_	.08	.08	.06	.05	.03	.15
SSRT _Shape_	.023	−.00	.00	−.01	.13	.16
**SSACC**_**Low Calorie**_	.15*	.08	.18*	.07	.20	.32*
SSRT _Low Calorie_	.16*	.07	.14^†^	.09	.37**	.13
**SSACC**_**High Calorie**_	.24***	−.01	.23**	−.07	.50***	.40**
SSRT _High Calorie_	.16*	−.03	.07	−.15	.41**	.26^†^
Depression _Eating_	.07	.14*	−.05	.10	.41**	.28*
Anxiety _Eating_	−.14*	−.01	−.19*	−.01	.05	−.06
Anger/Hostility _Eating_	−.05	−.05	−.09	−.08	−.17	−.11
Positive emotion _Eating_	−.22**	−.18**	−.24**	−.16*	.11	−.08

*Note*. Non- abdominal Obesity (*n* = 168, abdomen circumference ≤ 85 cm); Abdominal Obesity (*n* = 56, abdomen circumference ≥ 85 cm). SSACC = the stop signal accuracy. SSRT = the stop signal reaction time. ^†^ *p* < .10. *p < .05. **p < .01.***p < .001

### Partial correlations between anxiety and emotional eating

As presented in Table 3, overall trait anxiety and state anxiety was correlated with depression _eating_ and anger/hostility _eating_. Analyzing by non-abdominal obesity & abdominal obesity separately, partial correlations between trait anxiety, state anxiety and depression _eating_ were significant (*r* = .58, *p* < .001; *r* = 51, *p* < .001) for abdominal obesity female undergraduates. And partial correlations between trait anxiety and anxiety _eating_ were also significant (*r* = .33, *p* < .05). In a parallel analysis of the non-abdominal obesity group, only the correlation between trait anxiety and anger/hostility _eating_ was significant (*r* = .19, *p* < .05).

**Table 3:** Partial correlations between anxiety and emotional eating controlling for age, BMI, and family SES

Variables			Non-abdominal Obesity		Abdominal Obesity	
	Trait Anxiety	State Anxiety	Trait Anxiety	State Anxiety	Trait Anxiety	State Anxiety
Depression _Eating_	.23***	.14*	.07	−.01	.58***	.51***
Anxiety _Eating_	.16*	.12^†^	.13	.07	.33*	.27^†^
Anger/Hostility _Eating_	.23**	.14*	.19*	.12	.23^†^	.13
Positive emotion _Eating_	.02	.00	.08	.06	−.20	−.15

*Note*. Non- abdominal Obesity (*n* = 168, abdomen circumference ≤ 85 cm); Abdominal Obesity (*n* = 56, abdomen circumference ≥ 85 cm). ^†^ *p* < .10. *p < .05. **p < .01.***p < .001

### Mediation results

The structural equation model provided a good fit to the sample data with TLI = .98, CFI = 0.94, RMSEA = .00,χ^2^/*df* = .88. Cognitive difference of body image predicted the mediating variables trait anxiety and state anxiety (*β* = .38, 95% CI: 0.10–0.62, *p* < .01; *β* = .50, 95% CI: 0.21–0.74, *p* < .01). There was significant direct influence of cognitive difference of body image (*β* = .38, 95% CI: 0.05–0.71, *p* < .05), as the predicted variable, on depression _eating_. As predictive indirect effect of mediating variables in cognitive difference of body image on depression _eating_, only trait anxiety (*β* = .87, 95% CI: 0.03–0.21, *p* < .001) had indirect influences on the relationship between cognitive difference of body image and depression _eating_ in the female abdominal obesity group (see Fig. 1). There was significant direct influence of SES (*β* = .39, 95% CI: 0.14–0.61, *p* < .01), as the covariate, on depression _eating_.

## Discussion

The prevalence of abdominal obesity among Chinese adults was 37.4% (45.9% in females) according to the China Health and Nutrition Surveys in 2009 [62]. This study reported that the prevalence of abdominal obesity (defined as abdomen circumference ≥ 85 cm for females) was 25% for female college student, which showed a trend towards a lower prevalence of abdominal obesity compared to those in other studies [63]. There may be two aspects related to this difference in prevalence. On the one hand, the values of the reference standards were different. On the other hand, age ranges are different. The increase was larger among individuals between the ages of 40 and 59 years [62], which suggests that the prevalence of young female abdominal obesity among the subpopulation of 19–23 with a higher level of education was lower.

In this study, all the participants (100%) perceived themselves to be fatter than physical truth and wanted a more slender figure. Indeed, wanting a thinner image was a general trend. The differences and the AC-related differences in relation of inhibitory control, emotional eating, anxiety and body image between abdominal obesity and non-abdominal obesity in young women were compared. Abdominal obesity female youths and non-abdominal obesity ones showed statistically significant differences in body image (cognitive difference and expectational difference), anxiety (trait anxiety and state anxiety), which has already been seen in previous studies [36, 40]. In all SST measure variables, no statistically significant differences were found between two groups. These results are consistent with previous researches about female undergraduates motivated to manage weight and they found no correlation between SSRT in food trials, other any SST measure and BMI [64–66]. Contrary to our hypothesis, non-abdominal obesity female obtained higher scores in emotional eating variables, especially anxiety _eating_ and positive emotion _eating_. One reason may be that self-reported emotional eating is not related to greater food intake [67, 68]. People may be considerably biased in their ability to identify themselves as emotional eaters [69–71], whether they are fat or not. These results lead us to think that self-reported emotional eating measure may actually reflect eating concerns [70].

Interestingly, cognitive difference of body image have been associated with the SST, including SSACC _Low Calorie_, SSRT _Low Calorie_, SSACC _High Calorie_, and SSRT _High Calorie_ in all participants and non-abdominal obesity group. However, in the abdominal obesity group, the expectational difference of body image also positively associated with SSACC _Low Calorie_ and SSACC _High Calorie._ This means that food- specific inhibitory control variables, especially SSACC _High Calorie,_ only tends to be affected by expectational body image in abdominal obesity female groups. One possible explanation is that the abdominal obesity individuals desire to be thinner, but all the females often considered themself fatter than current body shape. One’s appearance is becoming increasingly important in modern society [72]. Hence, more normal body shape girls perceived themselves to be fatter than current body shape [73].

Results revealed that cognitive difference of body image is positively associated with emotional eating for female abdominal obesity individuals, but not for their non-abdominal obesity counterparts, in which cognitive difference of body image is negatively associated with emotional eating. Given prior findings linking body image dissatisfaction to obesity [74] and emotion to obesity risk [75], this study suggests the need for additional research on the potential mediating mechanisms linking these factors. In other words, one potential explanation for the discrepancy between the effects of cognitive difference of body image on emotional eating relates to whether these relationships are mediated by some other emotion. Emotional eating is more commonly reported by women than men [76], and it is often triggered by anxiety [77]. A potential mediating mechanism is anxiety given that social physique anxiety has been observed in individuals with body image dissatisfaction to obesity [78].

Our finding that emotional eating was linked to trait and state anxiety may reflect the fact that anxiety involves the avoidance motivational system and is subject to regulation by strategies such as eating [35]. These results are in keeping with those obtained in studies by other author [40]. The results of this study show that obese individuals high in negative affect may consume more calories in response to a negative mood compared to obese individuals low in negative affect, and to lean individuals. An important finding of this study was that trait and state anxiety mediated the relationship between cognitive difference of body image and depression _eating_ in female abdominal obesity individuals. Our studies could help elucidate the role of cognitive difference of body image in vulnerability to emotional eating among female abdominal obesity individuals with high anxiety.

This study has several limitations worth noting. First, the study was limited by its cross-sectional design and cause-effect relationships could not be established. Therefore, future studies using a longitudinal design will be required to clarify the direction of these associations. Second, emotional eating was collected from female youths using self-report questionnaires. It remains a possibility that people are unable to accurately report on their own emotional eating behaviour [79]. An alternative to self-report questionnaires as the criterion for emotional eating is to use a highly valid experimental food paradigm. Third, a convenient sample of students was used in the present study due to the complexity and particularity of the Food- inhibitory control testing process and the constraints of both financial and human resources. This sample was comprised purely of students from Xi’an of Shaanxi province, likely representing a collectivistic culture.

## Conclusion

Results demonstrated that anxiety mediated the relationship between cognitive difference of body image and depressed _eating_ in young females with abdominal obesity. In addition, only among abdominal obesity individuals, expectational difference of body image were significantly and positively correlated with food-specific inhibitory control and trait/state anxiety. The findings suggest it is of critical importance to promote a healthy body image recognition and expectation and improve mood regulation for young females with abdominal obesity high in trait anxiety.

## Data Availability

The data used during the current study are available from the corresponding author on reasonable request.

## Abbreviations

BMI: Body Mass Index
SMT: Silhouette-Matching Test
STAI: State-Trait Anxiety Inventory
SST: Stop Signal Task
EES: Emotional Eating Scale
AC: Abdomen Circumference
EDs: Eating Disorders
SSRT: Stop Signal Reaction Time
